# The Impact of Postpartum Depression on Quality of Life in Women After Child's Birth

**DOI:** 10.5812/ircmj.14995

**Published:** 2014-02-05

**Authors:** Zohreh Sadat, Masoumeh Abedzadeh-Kalahroudi, Mahboobeh Kafaei Atrian, Zahra Karimian, Zahra Sooki

**Affiliations:** 1 Trauma Nursing Research Center, Kashan University of Medical Sciences, Kashan, IR Iran; 2 Trauma Research Center, Kashan University of Medical Sciences, Kashan, IR Iran; 3 Department of Midwifery, Kashan University of Medical Sciences, Kashan, IR Iran; 4 Department of Reproductive Health, Shahroud University of Medical Sciences, Shahroud, IR Iran

**Keywords:** Depression Postpartum, Health, Mental Health

## Abstract

**Background:**

Postpartum depression (PPD) is a common problem after child's birth and may influence the quality of life (QOL). Investigation of postpartum QOL and depression can be useful for better care for mothers and improvement of their well-being.

**Objectives:**

The objective of this study was to assess the life quality in mothers with and without PPD.

**Patients and Methods:**

In a prospective study, women who had experienced child's birth with and without PPD were recruited in Kashan-Iran. PPD was measured using the Edinburgh Postnatal Depression Scale (EPDS) and QOL was measured by SF-36 questionnaire. Data collection was conducted at two assessment points: second month (n = 321) and fourth month (n = 300) postpartum. Based on EPDS, a score of 13 or more was defined as PPD. Mean scores of SF-36 questionnaire were compared between women with and without PPD at two assessment points and within each group from the first to the second assessments. Moreover, correlation between scores of EPDS and scores of life quality dimensions were evaluated. Data were analyzed by using the Student’s t-test, Mann–Whitney U-test, ANOVA, Kruskal-Wallis, Chi-square test, Pair t test, Wilcoxon, Pearson and Spearman Correlation Coefficient.

**Results:**

Differences in seven out of eight mean scores of QOL dimensions (except role-physical) between depressed and non-depressed women at the first and the second assessments were significant. Results of changes in mean scores of QOL dimensions from the first to the second assessments in each group showed that non-depressed women scored higher in all of eight dimensions with significant differences in two dimensions (bodily pain and role-emotional as well as mental health component). In depressed women, scores of life quality decreased in some of QOL dimensions but differences were not significant. There were significant negative correlations between EPDS scores and scores of seven out of eight SF-36 sub-scales (except role-physical) in addition to physical and mental health components at two assessments. The highest correlation was found between EPDS scores and emotional well-being and total scores of SF-36 dimension at the first and the second assessments (r = -o.489, r = -0.381), respectively.

**Conclusions:**

The findings demonstrated that postpartum depression leads to a lower life quality at second and fourth months postpartum. Integration of PPD screening into routine postnatal care is recommended.

## 1. Background

Several researches demonstrated that postpartum depression (PPD) is a common problem (1). In a study, the rate of PPD during six to eight weeks after delivery was 20.1 % (2). Incidence of postnatal depression has been reported between 0 % to nearly 60 % in a review of studies from different countries (3). The beginning of postpartum depression may be at the fourth week postpartum, although it is usually detected between sixth and twelfth weeks postpartum (4). The general symptoms of these women include depressed mood, weakness, disappointment, agitation, psychological distress, and sleep disorders (5). A number of changes are made during pregnancy and postpartum including organic, emotional, and societal alteration. Adjusting to these alterations may cause emotional problems in women after birth (6). The main predisposing factors for depression after delivery are history of mental disorders, depression during pregnancy, socio-economic insufficiency, and existence of other medical conditions (7-10). In addition, physical health problems after delivery are frequent and nearly 70 % of the mothers experience at least one problem in physical well-being due to birth. These problems are related to functional restrictions (11). Quality of life (QOL) after delivery is related to maternal social and demographic characteristics, birth factors, type of delivery, and social support (12, 13). Postnatal depression has serious impact on mothers that manifests as lower quality of life and inability to care of themselves, their partner, and infants (14, 15); moreover, occurrence of depression in these women is higher later in life (16). In a research, results showed there were significant correlation between postnatal depression and wellbeing condition (17, 18). In another study, occurrence of the PPD was 15 % and mothers with PPD had lower quality of life scores based on World Health Organization Quality of Life–Bref (WHOQOL-BREF) scale (19). Other researchers indicated that all domains of SF-36 QOL scale were lower significantly in mothers with PPD compare to mother without PPD; however, worse physical health condition was not related to intensity of depression (20). A study was performed by Barbosa et al. in Portuguese revealed that some of SF-36 sub-scales are impaired in women with PPD (21). Boyce et al. found inverse correlation between PPD and five out of eight domains of SF-36 scale (22). Although undesirable effects of PPD are well known, the influence of postnatal depression on quality of life or health status of mothers after birth has not been understood and few researches are done concerning this important issue (23).

## 2. Objectives

The Objective of the current study was to assess of QOL in mothers with and without PPD. The results of this study can be useful for better care for mothers and improvement of their well-being.

## 3. Materials and Methods

A prospective study was conducted from August 2007 to October 2008 on mothers who were referred to health centers for the prenatal care. Stratified random sampling was performed to select ten health centers. There are 25 health centers in Kashan city, Iran, that were divided into five geographical regions (north, south, west, east, and center). Two health centers were randomly selected from each region and based on the population covered; some samples were selected sequentially in the health centers. We have included all women with singleton pregnancy, term pregnancy, with a prenatal care started before 20 weeks gestation, uncomplicated pregnancies, not having depression during pregnancy based on EPDS score ≥ 13, and parity of 1 to 3. The study subjects had no other diseases or infertility history and were not divorced. Excluding criteria were women with instrumental deliveries, birth weight less than 2500 grams, dead child or fetus, child abnormality, no breast-feeding, and medical problems in child or mother. In the current study, 450 women were observed during the third trimester of pregnancy and 384 women of them met the inclusion and exclusion criteria. Overall, 365 pregnant women agreed to participate in the study. In the second month postpartum follow-up (the first assessment), 25 women were excluded from the study and four women did not return. The remaining 321 mothers completed depression and QOL questionnaires (246 and 75 mothers without and with depression, respectively). At the fourth month postpartum follow-up (the second assessment), 15 cases were excluded from the study (nine and six women without and with depression, respectively); six mothers did not retune (four cases of women without depression and two cases of women with depression). Finally, 300 mothers completed these questionnaires again (253 and 47 cases of women without and with depression, respectively). The size of the sample was chosen based on α = 5 %, β = 20 %, and the mean differences scores of QOL between women with and without depression according to the pilot study at second month postpartum. The mean differences scores of QOL between women with and without PPD were both 7.4. The standard deviations were 15.8 and 15.2 in women with and without PPD, respectively. Therefore, we needed 68 samples in each group that with considering PPD prevalence based on pilot study (23 %) and estimated 10 % loss in fallow up, we required nearly 365 samples in this cohort study.

The study approved by Ethics Committee of the Kashan University of Medical Sciences, the number code was 29/5/1/751 in 29/4/2007. Written informed consent was signed by mothers and trained midwifes informed them about the details of questionnaires and aim of the study. Women were assured that their information would be kept confidential and they can leave the study whenever they want. A structured questionnaire was used to assess women's socio-demographic and obstetric characteristics, which was completed by trained midwifes. Postpartum depression was assessed based on EPDS. The EPDS is a simple and extensively used instrument for screening postpartum depression symptoms; it consists of ten points and each point contains a four-item scale, with maximum scores of 30. A score of 13 or more displays a significant case of sever PPD, while borderline PPD detected by scores of 10 to 12 and zero to nine means non-depressed case (24). Reliability and validity of the EPDS in Iran was evaluated by Montazeri et al. (25). The QOL was measured by Iranian version of SF-36 questionnaire. SF-36 is a standard and well-known nonspecific health related QOL and showed to be highly practicable, reliable, and is an appropriate choice to evaluate QOL after delivery (26). The SF-36 in Iranian population is validated by Montazeri et al. (27). It consists of 36 items with eight sub-scales: Physical functioning (PF), role limitation due to physical problems or role-physical (RP), bodily pain (BP), general health (GH), vitality (VT), social functioning (SF), role limitation due to emotional problems or role-emotional (RE), and emotional well-being (EW). Each subscale ranged from zero to 100 with higher scores indicating a better condition. Furthermore, the SF-36 is divided into two components: Physical health (based on the PF, RP, BP, and GH scales) and mental health (relating to VT, SF, RE, and EW).

In the current study, QOL dimensions were assessed according to women’s socio-demographic characteristics at the first assessments. The mean scores of QOL dimensions were compared between women with and without PPD at two assessments. In addition, correlation between EPDS scores and scores of QOL sub-scales were assessed at two assessments points. The SPSS-16 software (SPSS Inc., Chicago, Illinois, the United States) was used for data analysis. Normal assumptions were checked by using One-Sample Kolmogorov-Smirnov Test and if it was necessary, nonparametric tests were used. Mean differences between groups were analyzed using the ANOVA, Kruskal-Wallis, Student's t-test, and Mann–Whitney U-test. The mean differences within groups between second and fourth months after birth were analyzed by Paired t test or Wilcoxon test. Moreover, nominal variables were analyzed by Chi-square test. The correlation between EPDS scores and scores of QOL sub-scales were evaluated by Pearson and Spearman Correlation Coefficient. P-value of less than 0.05 was regarded as statistically significant. The study protocol was approved by the Local Research Council in Kashan University of Medical Sciences.

## 4. Results

Of the 365 pregnant women that agreed to participate in the study, 321 mothers were followed at second month postpartum (the first assessment) and completed the EPDS and QOL questionnaires. At the fourth months postpartum (the second assessment), 300 mothers completed these questionnaires again. Based on EPDS questionnaire, 75 (23.36 %) and 47 (15.6 %) of mothers in the current study had sever PPD at second and fourth months postpartum, respectively. The participants' basic characteristics and the QOL at second month postpartum are presented in Table 1. Results showed that there were no significant differences between demographic variables and QOL mean scores. The relationship between PPD and mean scores of SF-36 dimensions at the first and the second assessments are presented in Tables 2 and 3, respectively. Our findings demonstrated that seven of eight SF-36 dimensions including both physical and mental health components scored significantly lower in the women with PPD in comparison to the women without PPD at the first and second assessments. Mean score in role-physical dimension was lower in women with PPD but the difference was not significant. In this study, changes of mean scores of QOL between second and fourth months postpartum were evaluated in each group. The QOL in women without PPD scored higher in all of the eight dimensions from the second to the fourth month postpartum and differences were significant in two dimensions (bodily pain and role-emotional as well as mental health component). In contrary, in women with PPD scores of life quality had increased in four out of eight QOL sub-scales and were scored lower in others sub-scales; however, differences were not significant in each of dimensions (Table 4). The correlation between EPDS scores and scores of SF-36 dimensions and total scores of physical and mental health components at the first and second assessments are presented in Table 5. There were negative correlations between EPDS scores and scores of all SF-36 sub-scales in addition to the physical and mental health components at the first and second assessments, except role-physical at the first assessment that its correlation with EPDS was not significant. The strongest correlation was found between EPDS scores and emotional well-being as well as total scores of SF-36 at the first and the second assessments (r = -o.489, r = -0.381) respectively.

**Table 1.** Basic Characteristics of 321 Mothers According to Quality of life 2 Months After Birth

Characteristics	No. (%)	Quality of Life, Mean ± SD	P value
**Age of mothers, y**	-	-	0.20^a^
< 30	268 (83.5)	59.14 ± 12.30	-
≥ 30	53 (16.5)	61.50 ± 11.32	-
**Child birth weight, gr**	-	-	0.51^b^
< 3000	93 (28.9)	60.73 ± 12.07	-
3000-3500	129 (40.2)	59.28 ± 11.73	-
≥ 3500	99 (30.9)	58.73 ± 12.81	-
**Number of childeren**	-	-	0.66^a^
1	153 (47.7)	59.23 ± 12.56	-
≥ 2	168 (52.3)	59.81 ± 11.80	-
**Type of delivery**	-	-	0.67^c^
Caesarean section	159 (49.5)	59.83 ± 13.52	-
Vaginal delivery	162 (51.5)	59.24 ± 10.68	-
**Occupational status**	-	-	0.96^a^
Employed	35 (10.9)	59.44 ± 13.90	-
Housewife	286 (89.1)	59.54 ± 11.95	-
**Unplaned pregnancy**	-	-	0.49^a^
No	280 (87.2)	59.71 ± 12.31	-
Yes	41 (12.8)	58.32 ± 11.11	-
**Undesired sex of baby**	-	-	0.65^c^
No	195 (60.7)	59.78± 12.14	-
Yes	126 (29.3)	59.15 ± 12.22	-
**Level of education**	-	-	0.10^d^
Primary school and illiterate	123 (38.3)	60.82 ± 11.49	-
Middle ans high school	157 (48.9)	58.06 ± 12.61	-
College education	41 (12.8)	61.32 ± 11.93	-
**Gender of baby**	-	-	0.61^c^
Male	164 (51.1)	59.82 ± 12.26	-
Female	147 (48.9)	59.23 ± 12.07	-

^a^ Student’s t-test was used

^b^ ANOVA test was used

^c^ Mann–Whitney U-test was used

^d^ Kruskal-Wallis test was used

**Table 2.** Scores of Quality of Life Dimensions According to Postpartum Depression at Second Month Postpartum

Dimensions	Postpartum Depression, No. (%)		P value ^a^
	No (n = 246)	Yes (n = 75)	
**Physical health**			
**Physical functioning**	58.63 (20.24)	52.82 (20.08)	0.030
**Role physical **	54.85 (18.16)	52.14 (18.73)	0.262
**Bodily pain**	60.34 (22.74)	50.09 (20.69)	0.001
**General health**	68.51 (16.71)	57.77 (16.71)	0.001
**Total physical health**	60.58 (14.36)	53.20 (14.15)	0.001
**Mental health**			
**Social functioning**	65.13 (21.87)	51.11 (19.33)	0.001
**Emotional well-being**	69.82 (18.90)	52.88 (17.94)	0.001
**Role emotional **	56.21 (22.07)	48.04 (20.60)	0.005
**Vitality**	60.18 (18.95)	53.54 (16.99)	0.007

^a^ Total mental health 62.83 (13.76) 51.39 (14.76) P < 0.001 should be added in a new row, Data analyzed by using student's t-test and Mann–Whitney U-test

**Table 3.** Scores of Quality of Life Dimensions According to Postpartum Depression at fourth Months Postpartum

Dimensions	Postpartum Depression, No. (%)		P value^a^
	No (n = 253)	Yes (n = 47)	
**Physical health**			
**Physical functioning**	58.05 (20.63)	51.21 (22.03)	0.04
**Role physical **	55.17 (19.19)	51.02 (20.66)	0.179
**Bodily pain**	63.73 (22.20)	52.07 (23.78)	0.001
**General health**	68.21 (16.80)	56.48 (18.38)	< 0.001
**Total physical health**	61.29 (13.50)	52.69 (14.45)	< 0.001
**Mental health**			
**Social functioning**	67.16 (20.99)	57.74 (17.92)	0.004
**Emotional well-being**	72.24 (18.12)	54.11 (17.28)	< 0.001
**Role emotional **	60.06 (21.46)	52.93 (20.49)	0.036
**Vitality**	61.02 (16.34)	51.26 (16.07)	< 0.001
**Total mental health**	65.12 (12.18)	54.01 (13.20)	< 0.001

^a^ Data analyzed by using Student's t-test and Mann–Whitney U-test

**Table 4.** Comparison of Mean Scores of Life Quality Dimensions Between second and fourth Months After Birth in women with and without postpartum depression in two-Time Assessments

Dimensions	Non-Depressed Women, No. (%) (n = 224)			Depressed Women, No. (%) (n = 38)		
	2 Months	4 Months	P value	2 Months	4 Months	P value^a^
**Physical health**						
**Physical functioning**	58.25 (20.23)	58.75 (20.29)	0.76	51.60 (19.38)	49.07 (22.27)	0.61
**Role physical **	55.32 (17.99)	56.07 (19.29)	0.66	51.44 (16.94)	49.88 (18.72)	0.70
**Bodily pain**	60.04 (22.89)	64.63 (22.57)	0.025	52.57 (20.38)	53.11 (18.35)	0.87
**General health**	68.22 (16.61)	69.41 (16.71)	0.31	56.10 (19.35)	55.04 (17.60)	0.74
**Total physical health**	60.46 (12.20)	62.21 (12.46)	0.07	52.91 (10.25)	51.77 (9.33)	0.52
**Mental health**						
**Social functioning**	65.47 (21.11)	67.52 (20.97)	0.22	49.98 (18.21)	52.42 (20.56)	0.61
**Emotional well-being**	70.27 (18.02)	73.11 (18.12)	0.05	52.73 (17.34)	55.21 (18.17)	0.42
**Role emotional **	56.06 (22.06)	60.45 (21.76)	0.028	51.87 (21.20)	52.27 (19.21)	0.93
**Vitality**	60.62 (16.24)	61.62 (18.31)	0.49	53.26 (17.79)	51.21 (16.85)	0.61
**Total mental health**	63.10 (13.08)	65.69 (10.66)	0.007	51.96 (11.98)	52.77 (10.69)	0.69

^a^ Data analyzed by using pair t test or wilcoxon

**Table 5.** The Correlation Between EPDS Scores and Dimensions of Quality of Life Scores second and fourth Months postpartum

Dimensions	Correlation		Correlation	
	2 months	P value	4 months	P value
**Physical health**				
**Physical functioning**	-0.124	0.022	-0.164	0.004
**Role physical **	-0.062	0.062	-0.142	0.013
**Bodily pain**	-0.237	< 0.001	-0.211	< 0.001
**General health**	-0.354	< 0.001	-0.266	< 0.001
**Total physical health**	-0.256	< 0.001	-0.290	< 0.001
**Mental health**				
**Social functioning**	-0.336	< 0.001	-0.132	0.020
**Emotional well-being**	-0.489	< 0.001	-0.361	< 0.001
**Role emotional **	-0.191	< 0.001	-0.186	0.001
**Vitality**	-0.221	< 0.001	-0.237	< 0.001
**Total mental health**	-0.432	< 0.001	-0.343	< 0.001
**Total QOL scores**	-0.419	< 0.001	-0.381	< 0.001

## 5. Discussion

The current study showed the important role of PPD on women's physical and mental health status after delivery. Our results demonstrated that QOL is impaired significantly in the women with PPD. To our knowledge, a few studies evaluated the role of PPD on QOL. Our results are consistent with some preceding studies. A research performed by Da Costa et al. on 78 Canadian women at fourth through thirty-eighth weeks postpartum showed that based on EPDS ≥ 10, scores of all SF-36 sub-scales, including physical and mental health components, were lower in women with PPD in comparison to normative data in Canadian women (20). In our study, seven out of eight dimensions were scored lower in women with PPD; however, we had defined PPD based on EPDS score ≥ 13. Moreover, we compare the women with PPD to the women without PPD. Consistent with our findings, these authors showed decreased scores in mental and physical health components of SF-36 in women with PPD. A study was performed by Barbosa et al. with the aim of determining QOL in adolescence women with PPD (14-20 years). A total of 177 adolescents were interviewed between sixth and twentieth weeks postpartum; based on EPDS, PPD was found in 61 (34.5 %) participants. Barbosa et al. reported the low scores on all sub-scales of the SF -36 in adolescents with PPD, but differences were statistically significant in the following areas: mental health, vitality, general health, social and physical functioning. There was no significant differences for three areas consisted of role-physical, role-emotional, and bodily pain (21). Nevertheless, in our study QOL was assessed in women aged 20-40 years. In another study by Rojas et al. on 159 women up to eleventh month postpartum, significant impairment in emotional and physical health in addition to the vitality of the SF-36 sub-scales was seen in women with PPD in comparison to the women without PPD (28). However, our study was performed at second and fourth months postpartum.

De Tychey et al. conducted a study in 181 French mothers during first two months postpartum with the aim of assessing the influence of PPD on the QOL as well as the impact of child’s sex on PPD and QOL. Finding showed that in mothers with PPD all SF-36 sub-scales, including physical and mental components, were impaired severely. Furthermore, having a male infant decreased QOL scores and increased occurrence of PPD (29). Boyce et al. reported that five out of eight dimensions of SF-36 were impaired in Australian women with PPD at 24th week postpartum, in this study, there was no significant differences between PPD and three dimensions of SF-36, namely, physical function, bodily pain, and general health (22). A cross sectional study was conducted in the United States on two groups of women with and without PPD at sixth through 26th weeks postpartum each consisted of 23 women. The aim of this study was to compare functional statues between women with and without PPD. Functional statues was defined by several items including personal care, care of infant, care of household, job activity, and social activity. Results of this study showed that personal care, household care, and social functioning were lower in women with PPD but there was no difference in care of infant (4). In Another research in Brazil, QOL and PPD were assessed on postpartum mothers. In this study, QOL questionnaires consisted of WHOQOL-Bref and Multicultural Quality of Life Index (MQLI); PPD questionnaires were the Postpartum Depression Screening Scale (PDSS) and EPDS. They demonstrated significant negative correlations between total scores of QOL and PPD. Correlations between scores of EPDS and two QOL tools were 0.71 and 0.73, respectively (30). A study performed by Choi et al. on 130 Korean women between first to third weeks postpartum aimed to investigate the influence of Fatigue and PPD on QOL in postpartum women. In this study, PPD was defined by EPDS and QOL was defined by Maternal Postpartum Quality of Life Questionnaire (MPP-QOL) that assesses QOL during the early period after birth. Findings of this study showed a negative correlation between mean scores of fatigue and PPD with QOL (r = -0.44, P < 0.01, r = -0.42), respectively. Moreover, a positive correlation was observed between fatigue and PPD (r = 0.59, P < 0.001) (31). In a study by Yeo et al. on 211 women during first to third weeks postpartum PPD and QOL were assessed. The QOL was defined by SmithKline Beecham Quality of Life (SBQOL) questionnaire. Findings of this research demonstrated a significant negative correlation between PPD and QOL (r = -0.62 P < 0.001) and a positive correlation between child's birth experience and QOL (r = 0.43 P < 0.001) (32). Zubaran et al. in Southern Brazil investigated 101 mothers between second to twelfth weeks postpartum; they assessed the correlation between PPD and QOL. The PPD was assessed by PDSS and EPDS. Health statues was assessed by the Personal Health Scale (PHS) and the 12-item General Health Scale (GHS). Results of this study showed that there was a significant negative correlation between health status and PPD (r = -0.74 and r = -0.79 for correlation between EPDS scores and scores of PHS and GHS, respectively) (17). Although in above studies QOL was not evaluated by SF-36 instrument, these results were consistent with our study results that showed a negative correlations between scores of PPD and total scores of QOL at the first and second assessments (r = -0.419 and r = -0.381, respectively).

The findings of our study suggest that PPD severely decreased all domains of life quality except the role-physical at second and fourth months postpartum and led to lower physical and mental health components at two times assessments. Anyway, the estimated difference in scores of QOL may be confounded by depression during pregnancy. We realize that, our study is limited by the absence of data on prenatal depression and prenatal QOL. Another limitation of this study was using a general instrument for assessing QOL. However, our research was a prospective study using a special instrument for PPD (namely, EPDS) and the study population was ethnically a uniform group. Investigation of postpartum QOL and related factors such as PPD can be useful for prevention programs and care of postpartum mothers; hence, integration of PPD screening into routine postnatal care is recommended.
